# HPLC-Analysis, Biological Activities and Characterization of Action Mode of Saudi *Marrubium vulgare* against Foodborne Diseases Bacteria

**DOI:** 10.3390/molecules26175112

**Published:** 2021-08-24

**Authors:** Mayasar Al-Zaban, Souheila Naghmouchi, Nada K. AlHarbi

**Affiliations:** 1 Biology Department, College of Science, Princess Nourah Bint Abdulrahman University, Riyadh 11671, Saudi Arabia; n.souheila21@yahoo.com (S.N.); nkalharbi@pnu.edu.sa (N.K.A.); 2 National Research Institute of Rural Engineering, Water and Forestry, University of Tunis Carthage, Street of Hedi Karay BP N 10, Ariana 2080, Tunisia

**Keywords:** antioxidant activity, antibacterial activity, *Marrubium vulgare*, phenolic content, methanolic extract

## Abstract

The present study aims to evaluate the chemical composition, metabolites secondary and pharmacology activities of methanolic extract of *Marrubium vulgare* collected from King Saudi Arabia. Moreover, the primary mode of action of the tested extract was studied here for the first time against *E. coli* and *L. monocytogenes.* HPLC analysis shows that the major components in the tested extract are luteolin-7-*O*-d-glucoside, ferulic acid and premarrubiin. Obtained data demonstrated that the investigated extract was richer in phenol (26.8 ± 0.01 mg/GAE g) than in flavonoids (0.61 ± 0.05 mg EC/mL). In addition, the methanolic extract showed an important antioxidant capacity against the DPPH (IC_50_ = 35 ± 0.01 µg/mL) and ABTS (IC_50_ = 25 ± 0.2 µg/mL) radical scavenging and a strong inhibition of acetylcholinesterase enzyme with an IC_50_ value corresponding to 0.4 mg/mL. The antibacterial activity demonstrated that the evaluated extract had significant activity against both Gram-positive and Gram-negative bacteria. The effect of time on cell integrity on *E. coli* and *L. monocytogenes* determined by time–kill and bacteriolysis tests showed that the *M. vulgare* extract reduced the viability of both strains after 8 and 10 h and had a bacteriolytic effect against two different categories of bacteria, Gram-positive and negative, which are not of the same potency. Based on obtained data, it can be concluded that Saudi *M. vulgare* has a high pharmacological importance and can be used in preparation of food or drugs.

## 1. Introduction

The plant kingdom is an important source of natural remedies for different diseases. Due to their potential activities, the valorization of medicinal plants used by popular tradition is becoming very interesting [1]. Diverse investigations are in progress to find new functional ingredients from medicinal plants and to inhibit the growth of causal agents of foodborne diseases and food spoilage, particularly *Escherichia* *coli*, *Salmonella* spp., *Staphylococcus aureus* and *Bacillus* spp. [2,3]. Generally, there are three types of food preservatives: inorganic compounds, organic compounds and natural food preservatives. Given that the risk and stability of inorganic and organic compounds are increasing [4], it has, thus, become necessary to develop a natural antimicrobial agent. 

The genus *Marrubium* of the Lamiaceae family comprises forty-eight species and has been previously studied for their biological activities. Among them, *Marrubium vulgare* L. is one of medicinal plants that is grown and distributed in Saudi Arabia and also is a very common plant in North Africa, Central and West Asia and Southern Europe [5]. It has been used in traditional medicine for various purposes. It has been proved to possess several properties such as antibacterial, antioxidant [6,7,8], antidiabetic [9], anti-inflammatory [10], anti-hypertensive [11], vasorelaxant [12], hypoglycemic [13], cytotoxicity [14] and anti-cancer [15] activities.

The phytochemical analysis demonstrated that *M. vulgare* was rich in different compounds such as polyphenols, tannins, flavonoids, diterpenes and saponins [16,17], which justifies their biological activities. Despite there being several studies concerning the chemical composition and biological activities of *M. vulgare* extract, according to our knowledge, there is no available research which has focused on the action mode of *M. vulgare* against bacteria. In addition, the biological activities, in particular the anti-acethycolinesterase power of this plant harvested in Saudi, have been scarcely investigated. Therefore, the aims of the current investigation were, firstly, to determine the contents of the secondary metabolites in *M. vulgare* methanolic extract harvested in Saudi Arabia and its anti-acethycolinesterase and antioxidant activities using two tests (DPPH radical scavenging capacity and ABTS radical cations). Then, the antibacterial activity was evaluated against nine different bacteria species and the characterization of the action mode against *E. coli* and *L. monocytogenes* was also assessed by time–kill and Lysis assays. 

## 2. Results and Discussion 

Given that the aqueous extract of *M. vulgare* was not active against bacteria and the acethycolinesterase enzyme (data not shown), we were limited in the current study to the determination of the chemical composition and biological activities of the methanolic extract of *M. vulgare*. In fact, we observed that the extraction with methanol was better than the extraction with water.

### 2.1. HPLC Analysis

**Figure 1:**
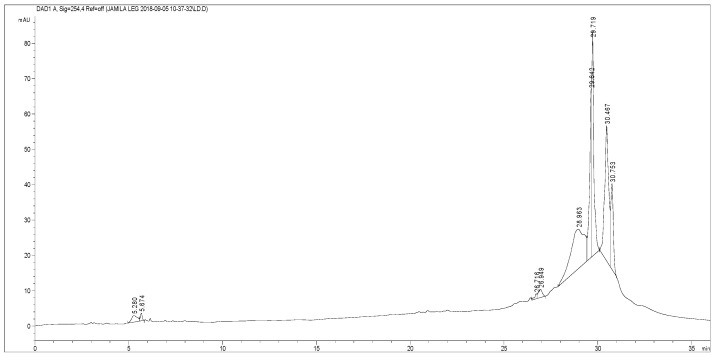
HPLC chromatogram of methanolic extract of *M. vulgare*.

The HPLC analyses of *M. vulgare* methanolic extract were summarized in Table 1. Identification was possible by a comparison with the retention time and UV spectra of the phenolic chromatogram of the fraction with those of pure standards (Figure 1). The obtained data revealed the presence of eight polyphenolic compounds in Saudi *M. vulgare* methanolic extract: premarrubiin, luteolin-7-*O*-d-glucoside, ferulic acid, terniflorin, cirsimaritin, amentoflavone, marruboside and gallic acid. The dominant compounds were ferulic acid (28.9 mg/g), luteolin-7-*O*-d-glucoside (26.35 mg/g) and premarrubiin (28.28 mg/g). The majorities of these compounds have been previously identified in some *Marrubium* species. For example, Wojdyłoa et al. [18] demonstrated that Polish *M. vulgare* extract contains ferulic acid. In addition, Rezgui et al. [19] showed that the main phenolic compounds in Tunisian *M. vulgare* extract are sinapic acid, quercetin, ferulic acid, p-coumaric acid, caffeic acid, apigenin and luteolin. 

The constituents observed in *M. vulgare* extract are well-known as bioactive compounds with antioxidant, antimicrobial, anti-inflammatory and analgesic activities. This evidence justifies the traditional and popular use of their aerial parts.

### 2.2. Secondary Metabolites Contents

The total polyphenols and flavonoid contents determined using colorimetric dosage showed that Saudi *M. vulgare* methanolic extract was richer in phenol than flavonoid. The total phenol concentration was 36.8 ± 0.01 mg gallic acid per gram (GAE/g), whereas the total flavonoid content was 1.61 ± 0.05 mg catechin equivalents per ml (CE/mL). These contents were comparable to those described in the literature for the same species (Table 2). In fact, Aouadhi et al. [8] demonstrated that the contents of polyphenols and flavonoids in Tunisian *M. vulgare* extract were 26.8 mg GAE/g and 0.61 mg CE/g, respectively. The means of phenolic and flavonoids contents recorded for Moroccan *M. vulgare* extract were 60.409 mg GAE/g and 33.81 mg CE/g, respectively [20]. Okur et al. [21] signaled that the German extract showed 48.97 ± 0.82 mg GA/g corresponding to the total phenolic amounts. In the same way, Ouchemoukh et al. [22] showed that the Algerian *M. vulgare* exhibited an important content of total phenolics and flavonoid, equivalent to 40 mg GAE/g and 10 mg/g, respectively, whereas Amri et al. [7] reported that the *M. vulgare* extract was richer in flavonoid (45.21 mg/g) than in polyphenols (6 mg GAE/g). 

The high concentrations of secondary metabolites in this species may contribute to important biological activities of *M. vulgare*. The obtained data justify the use of the evaluated plant overall its distribution area in the world by ancient and actual local human populations. Sengul et al. [23] showed that phenolic compounds exhibit considerable free radical scavenging activities and metal ion-chelating properties, preventing metal-induced free radical formation. In the same way, Gulcin et al. [24] signaled that polyphenolic compounds seem to have an important role in stabilizing lipid oxidation and be associated with antioxidant activity.

### 2.3. Antioxidant Activity

Two in vitro methods (DPPH and ABTS radical scavenging activities) were assessed to evaluate the antioxidant activity of *M. vulgare* methanolic extract. According to DPPH and ABTS tests, it could be concluded that the Saudi *M. vulgare* methanolic extract showed important antioxidant activity being close to control positive (Butylated hydroxytoluene (BHT)) with IC_50_ = 32 µg/mL (DPPH), 21 µg/mL (ABTS) and IC_50_ = 30 µg/mL (DPPH), 20 µg/mL (ABTS), respectively. The obtained data were agreed with previous studies showing a higher antioxidant activity of *M. vulgare* methanolic extract. For example, the IC_50_ of Tunisian *M. vulgare* extract in the presence of DPPH was 35 µg/mL and 38 µg/mL reported by Aouadhi et al. [8] and Amri et al. [7], respectively. In addition, Bouterfas et al. [25] showed wide ranges of IC_50_ (DPPH) varying from 33.7 to 124 µg/mL of Algerian *M. vulgare* methanolic extract collected from three sites, while the lowest antioxidant power was observed in Moroccan and German *M. vulgare* methanoIic extracts where the IC_50_ was 2.4 mg/L and 0.8 mg/L and 2.08 and 1.33 mg/mL in the presence of DPPH and ABTS, respectively [20,21]. 

The variation of antioxidant activities according to the studies may be related to the observed variation in phenolic and flavonoids contents. Numerous investigations reported the presence of significant linear correlations between the values for the concentration of phenolic compounds and the antioxidant activity of plant samples [18,26], indicating that extracts with highest polyphenol contents show higher antioxidant activity. However, based on the data presented in Table 2, it can be concluded that a significant correlation between antioxidant power and polyphenol count does not exist and there are other compounds that may be responsible for the antioxidant activity of the *M. vulgare* extract.

Generally, variability in metabolites contents and antioxidant activities may be related to different geographical sources, the harvesting seasons, the genotype, the climate, the soil composition, the drying procedure and the distilled part of the plant. Many investigations verified that the change in the antioxidant activity of the methanolic extract of *M. vulgare* was due to the sampling locality, soil and climatic variations [25,27].

### 2.4. In Vitro Acetylcholinesterase Inhibition

Acetylcholinesterase is an enzyme able to hydrolysis acetylcholine into acetic acid and choline that serves as a transmitter substance of nerve impulses through synapses [28]. The abundance of acetylcholinesterase is responsible to Alzheimer’s disease [29]. Therefore, the main appreciated drugs for Alzheimer’s disease must contain acetylcholinesterase inhibitors. In the current investigation, *M. vulgare* methanolic extract was tested for its anti-acetylcholinesterase activity. Obtained data showed that the tested extract inhibited acetylcholinesterase activity with IC_50_ values correspond to 0.4 mg/mL. This result was better than those reported by Orhan et al. [30], Ouchemoukh et al. [22] and Salaj et al. [31] for the standard galantamine (1 mg/mL), Algerian *M. vulgare* (0.52 mg/mL) and Serbian *M. vulgare* (2.281 mg/mL), respectively (Table 3). In the same way, Vladimir et al. [32] showed that 1mg/mL of ethanolic extract of *Marrubium incanum* did not achieve a 50% inhibition of enzyme activity. However, IC_50_ values (0.277 mg/mL) of *Marrubium desertia* methanolic extract were higher than those observed in the current study [33]. In addition, Orhan et al. [34] showed that the acetone extract of *M. vulgare* displayed an important anti-acetylcholinesterase activity reaching 76.30 ± 0.18% of inhibition at 100 µg/mL. 

The important anti-acetylcholinesterase activity obtained with *M. vulgare* extract could be attributed to its secondary metabolite contents. A previous investigation signaled that flavonoids and other phenolic compounds possess anti-acetylcholinesterase activity [22,30].

### 2.5. Antibacterial Activity

The results of the antibacterial assays of *M. vulgare* methanolic extract against nine bacteria are summarized in Table 4. The obtained data indicated that the investigated methanolic extract displayed an important antibacterial power with the inhibition zone and minimum inhibition concentration (MIC) varying from 13 to 17 mm and 6.25 to 25 mg/mL, respectively. In addition, a variation effect according to the tested microbial strains was observed. The highest antibacterial activity observed against *K. pneumoniae* with MIC was 6.25 mg/mL. The lowest activity recorded for *B. cereus*, *L. monocytogens*, *S. aureus*, *P. aeruginosa* and *S. typhimurium* with MIC was 25 mg/mL. 

Obtained data were partially in agreement with previous studies with the same plant harvested in Tunisia and Morocco. *M. vulgare* methanolic extract exhibited antibacterial activity but there were often large variations in its degree against Gram-negative and Gram-positive bacteria. For example, Kanyonga et al. [35] reported that *M. vulgare* extract collected from Morocco posed important antibacterial activity against *B. subtilis*, *S. epidermidis* and *S. aureus* (MIC = 100 mg/mL) and moderately effective against *P. vulgaris* and *E. coli* (MIC = 400 mg/mL), while ineffective in the case of *P. aeruginosa*. Moreover, Aouadhi et al. [8] showed that Tunisian *M. vulgare* extract was efficient against both Gram-positive (*B. cereus*, *L. monocytogens*, *S. aureus*) and Gram-negative bacteria (*E. coli*, *P. aeruginosa*, *A. hydrophila*, *S. typhimurium)* with MIC ranging from 12.5 to 25 mg/mL. 

Based on these results, it can be concluded that the M. vulgare methanolic extract had in vitro antibacterial activity but did not have a selective effect on the basis of the cell wall differences of bacterial microorganisms. Further studies will be necessary to understand the mechanisms of action underlying the effects of the extract.

### 2.6. Characterization of Action Mode of Methanolic Extract 

The characterization of the mechanisms of action of *M. vulgare* methanolic extract was carried out here for the first time. In fact, experiments on cell death and bacteriolysis of the evaluated extract against two bacterial species (*E. coli* and *L. monocytogenes*) were used to measure the effects induced by time-dependent treatments on cell viability.

#### 2.6.1. Dynamics Action of Methanolic Extract: Kill–Time Analysis 

**Figure 2:**
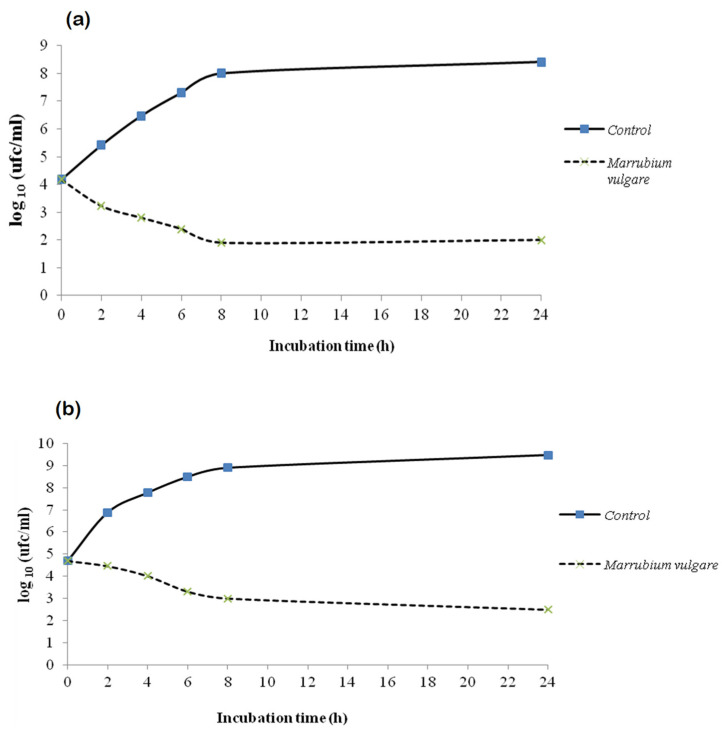
Time–kill curves *E. coli* (**a**) and *L. monocytogenes* (**b**) cultures untreated and treated with the methanolic extract of *M. vulgare* at concentration corresponding to MIC.

In order to study the antibacterial action mode of the tested extract, the growth of two selected bacteria (*L. monocytogenes* and *E. coli*) were monitored in the absence and presence of the extract at a concentration corresponding to MIC over a period of 24 h (Figure 2). As presented in Figure 2, the control population showed a classic growth curve with three phases. First, the exponential phase of the growth began in the first two hours where bacteria multiplied rapidly because nutrients were in abundance. Then, the stationary phase and the beginning of the decline phase took place between 8 and 24 h, the supply of nutrients becoming limited. It was the environmental and trophic factors that limited the growth of a bacterial culture and the death of bacteria compensates for their multiplication. 

In the presence of the extract at a concentration corresponding to MIC, the shape of the growth curve was reversed and the three phases no longer appeared indicating the cessation of growth of both bacterial strains tested after incubation for 24 h at 37 °C. In fact, within two hours of treatment, the number of viable cells decreased. It reached the limit of detection (inhibition of about 50% of the initial population = 2 log (CFU/mL)) after 8 h and 10 h for *E. coli* and *L. monocytogenes,* respectively. Based on these results, it can be signaled that the bactericidal effect of *M. vulgare* methanolic extract was time dependent and there was a difference in the mode of action of tested extract against Gram-positive (*L. monocytogenes*) and Gram-negative bacteria (*E. coli*).

#### 2.6.2. Determination of the Lytic Action of Methanolic Extract 

In order to determine the lytic action of Saudi *M. vulgare* methanolic extract on two bacterial species (*L. monocytogenes* and *E. coli*), we measured the absorbance of the bacterial strains in the absence and presence of the extract at a concentration corresponding to MIC. The loss of absorbance after 2 h of incubation was evaluated based on the initial absorbance. The results were, therefore, expressed as the ratio of the absorbance measured at time T to the absorbance at 620 nm measured at time zero ((OD_620_ (T)/OD_620_ (T_0_)) × 100).

**Figure 3:**
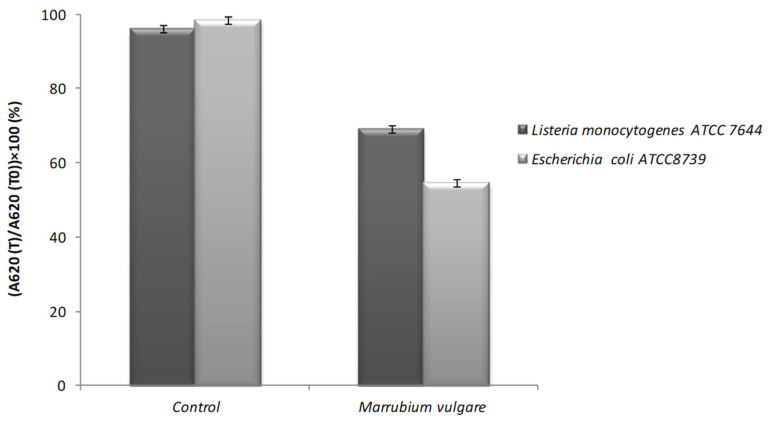
Cell integrity of *E. coli* (grey bars) and *L. monocytogenes* (black bars) after treatment by methanolic extract of *M. vulgare* at concentration corresponding to MIC. The results are expressed as the ratio of the absorbance measured at time T to the absorbance at 620 nm measured at time zero ((OD_620_ (T)/OD_620_ (T_0_)) × 100).

Figure 3 shows that in the case of the control (without extract), the absorbance of two bacterial strains was around 100% indicating the absence of cell lysis. However, the addition of extract caused a decrease in the initial absorbance of both bacteria. Indeed, the optical density decreased to 50% and 70% for *E. coli* and *L. monocytogenes*, respectively.

Usually, some antimicrobial agents destroy the bacterial membrane, irreversibly leading to cell death by a lytic process [36,37,38,39]. Indeed, the obtained data confirmed the kill–time assay when showing that the methanolic extract of *M. vulgare* had a bacteriolytic effect against two different categories of bacteria, Gram positive and Gram negative, which were not in the same potency. Indeed, *E. coli* was the more sensitive to the effect tested extract than *L. monocytogenes*. These results are consistent with those of Horne et al. [40] who showed that the essential oils of oregano, rosewood and thyme generate lytic had effects on *Streptococcus pneumoniae*. However, other authors have reported that plant extracts do not lyse bacterial cells but compromise the structural integrity of the plasma membrane and induce the loss of cytoplasm material [41].

## 3. Material and Methods 

### 3.1. Plant Material

The plant collection was done after permission from the scientific research committee at Princess Nourah bint Abdulrahman University, in accordance with national guidelines. The plant material (leaves of *M. vulgare)* used in this study was collected from Wadi Kama, Al-Taif governorate, Saudi Arabia in November 2020. The plant was botanically identified according to the “Flora of The kingdom of Saudi” [42]. A voucher specimen was deposited in the microbiology laboratory of the College of Sciences in Princess Nourah bint Abdulrahman University. The use of plant parts in the present study complied with national guidelines.

### 3.2. Preparation of Methanolic Extract 

The air-dried leaves were finely ground with blade-carbide grinding. A total of 10 mL of pure methanol (80%) was used to extract 1 g of *M. vulgare* leaves. The extract was mixed for 30 min, kept for 24 h at 4 °C. Then, it filtered through a Whatman No. 4 filter paper, evaporated under vacuum to dryness and was stored at 4 °C until analyses [43]. 

### 3.3. HPLC Analysis 

The separation of phenolic compounds was performed using an Agilent 1100 series HPLC system equipped with an in-line degasser (G 1322A), a quaternary pump (G 1311A), a thermostatic automatic sampler (G 1313A), a column heater (G 1316A) and a diode array detector (G 1315A). Instrument control and data analysis were performed using Agilent HPLC ChemStation 10.1 edition under Windows 2000. The separation was carried out on an ODS C18 column in the reverse phase (4 mm, 250 × 4.6 mm, Hypersil) used as a stationary phase at room temperature. The mobile phase consisted of acetonitrile (solvent A) and water with acetic acid as solvent B (0.2 mL/100 mL water). The flow rate was maintained at 0.5 mL/min. The gradient program was as follows: 15% A/85% B 0–12 min, 40% A/60% B 12–14 min, 60% A/40% B 14–18 min, 80% A/20% B 18–20 min, 90% A/10% B 20–24 min, 100% A 24–28 min. The injection volume was 20 μL and the peaks were monitored at 280 nm. Peak identification was obtained by comparing the retention time and UV spectra of the phenolic chromatogram of the fraction with those of pure standards purchased from Sigma (St. Louis, MO, USA).

### 3.4. Secondary Metabolites Content 

#### 3.4.1. Determination of Total Phenolic Content 

The method previously reported by Slinkard and Singleton [44] was employed to determine the phenolic content of tested extract using Folin–Ciocalteu reagent. The obtained data were expressed as mg gallic acid equivalents per gram dry weight (GAE/g DW) through the calibration curve with gallic acid.

#### 3.4.2. Determination of Total Flavonoid Content

The aluminum chloride colorimetric method was utilized to measure the total flavonoid content [45]. An aliquot (1 mL) of 2% AlCl_3_ methanolic solution was mixed with 1 mL of evaluated extract. The absorbance was measured at 430 nm in a Shimadzu 160-UV (Tokyo, Japan) spectrophotometer, after incubation at room temperature for 15 min. The results were given as rutin equivalent per ml extract (RE/mL extract). 

### 3.5. Antioxidant Activity of Methanolic Extract 

#### 3.5.1. Free Radical Scavenging Activity Using DPPH 

The method signaled by Hatano et al. [46] was used to estimate the DPPH radical scavenging capacity of tested extract. In fact, 0.5 mL of 0.2 mM DPPH methanolic solution was added to 1 mL of methanolic extract. The reaction was allowed to stand at room temperature in the dark for 30 min and the absorbance was recorded at 517 nm against a blank (methanol solution). The ability to scavenge the DPPH radical was calculated using the following equation: scavenging effect (%) = [(A_0_ − A_1_)/A_0_] × 100, where A_0_ and A_1_ are the absorbance of the control and the sample, respectively. 

#### 3.5.2. Free Radical Scavenging Ability Using ABTS Radical Cation 

ABTS radical cation (ABTS^+^) was produced by reacting ABTS stock solution with 2.45 mM potassium persulfate and keeping the mixture at room temperature for 24 h before use in the dark [47]. The percentage inhibition of the ABTS cation radical by the samples was calculated according to the following formula: scavenging effect (%) *=* [(A_0_ − A_1_)/A_0_] × 100, where A_0_ is the absorbance of the blank sample and A_1_ is the absorbance of the sample. 

### 3.6. Acetylcholinesterase Inhibition

Ellman method was used to estimate AChE enzymatic activity of tested extract according to Khadhri et al. [48]. The percentage inhibition of acetylcholinesterase by the samples was calculated by the following formula: *I*(%) = 100 − [(*V*_sample_/*V*_control_) × 100], where A_sample_ represents the absorbance of the sample and A_control_ is the absorbance without the sample. IC_50_ indicates the extract concentration providing 50% inhibition. It was defined by plotting the inhibition percentage opposed to the solution concentrations.

### 3.7. Evaluation of Antimicrobial Activity 

The antibacterial power of *M. vulgare* methanolic extract against nine pathogens bacteria representatives of Gram-positive (*Staphylococcus aureus* ATCC 6538, *Bacillus cereus* ATCC 1247, *Listeria monocytogenes* ATCC 7644, *Enterococcus faecalis*) and Gram negative (*Escherichia coli* ATCC 8739, *Pseudomonas aeruginosa* ATCC 9027, *Salmonella arizona* ATCC25922, *Salmonella typhimurium* NCTC 6017, *Klebsiella pneumoniae*) were determined using two methods.

Firstly, the disc diffusion method was used to evaluate the qualitative antibacterial activity of *M. vulgare* methanolic [49]. In fact, 100 μL of each bacterial species (10^8^ CFU/mL) was spread on Muller Hinton agar plates. Sterile filter paper discs (6 mm in diameter) were separately impregnated with 15 μL of tested extract and placed on the agar which had previously been inoculated with the selected bacteria. Gentamicin (10 µg/disc) was used as a positive reference. Negative control corresponded to disc without sample. To determine the solvent activity, solvent control disc was employed. The inoculated plates were incubated for 24 h at 37 °C. The diameter of the growth-inhibition zone (including disc diameter of 6 mm) was used to estimate the qualitative antimicrobial activity of tested extract.

The quantitative antibacterial activity of *M. vulgare* extract was evaluated by determining minimum inhibitory concentrations (MIC) and minimum bactericidal concentrations (MBC). For that, broth dilution method as described by Cosentino et al. [50] and modified by Aouadhi et al. [8] was assessed. Microbial growth was indicated by the presence of turbidity and a ‘pellet’ on the tube bottom. MIC was recorded visually as the lowest concentration in each row that completely inhibited bacterial growth. MBC is usually an extension from MIC, where the micro-organisms quantitatively indicated that the minimum concentration had no viable organisms appear in the culture [8].

### 3.8. Primary Mode of Action of Methanolic Extract 

The mode of action of *M. vulgare* methanolic extract was assessed by determining the effect of time on cell integrity using time–kill studies and bacteriolysis assay. This experiment was realized against two micro-organisms representative of Gram-negative and positive bacteria (*E. coli* and *L. monocytogenes*, respectively).

#### 3.8.1. Time–Kill Studies

Time–kill studies allow characterizing the antibacterial activity of tested extract by evaluating the reduction in bacteria count in presence of extract at their MIC over several hours. In fact, in the current study, the method described by Klepser et al. [51] and modified by Viljoen et al. [52] was used to evaluate the effect of *M. vulgare* methanolic extract against two representing Gram-positive and negative bacteria. Activities of tested product used at their MIC were evaluated against *E. coli* and *L. monocytogenes* by measuring the reduction in the number of CFU/mL over 24 h. The limit of quantification by this method was 10^2^ CFU [9].

#### 3.8.2. Bacteriolysis

The bacteriolysis assays of tested extract against *L. monocytogenes* and *E. coli* were assessed according to the standard method described by Guinoiseau et al. [53] and Carson et al. [54]. The results were expressed as a ratio (in percent) of the OD_620_ at each time point versus the OD_620_ at 0 min.

### 3.9. Statistical Analysis

All the results are expressed as mean ± standard deviation of three replications. The data were processed using Microsoft Excel 2007. 

## 4. Conclusions 

This study provided the first investigation on biological activity of *M. vulgare* methanolic extract and the characterization of the action mode against two bacterial species in Saudi. This extract was richer in phenol than in flavonoids. The results of the antioxidant activities, evaluated by two different methods, pointed out strong protective activity against the scavenging of DPPH and ABTS free radicals. In addition, the tested extract had significant anti-acetylcholinesterase and antibacterial capacities. The characterization of action showed that the tested extract had a bacteriolytic effect against two different categories of bacteria, Gram-positive and Gram-negative, which were not of the same potency. All those results valorize *M. vulgare* as a medicinal plant which can be a source of biological active compounds. Thus, this species might be a good candidate for further investigation in developing a new antioxidant and antimicrobial. It can be used as a natural additive in food, cosmetic and pharmaceutical industries instead of more toxic synthetic compounds.

## Figures and Tables

**Table 1:** Chemical identification by HPLC of methanolic extract of *Marrubium vulgare*.

Retention Time (min)	Compounds	Concentration (mg/g of Dry Weight)
4.75	Gallic acid	0.74
5.28	Marruboside	1.92
26.72	Amentoflavone	0.61
26.88	Cirsimaritin	1.67
28.69	Ferulic acid	28.90
29.67	Luteolin-7-*O*-d-glucoside	26.35
30.47	Premarrubiin	28.28
30.76	Terniflorin	11.52

Quantification was carried out by the external standard method from integrated peak areas of samples at 280 nm (UV absorption maximum of flavanone glycosides).

**Table 2:** Total polyphenol content and antioxidant activity of methanolic extract of *M. vulgare* reported in different studies.

Origins	DPPH (IC_50_) (mg/mL)	ABTS (IC_50_) (mg/mL)	Total Polyphenol (mg GAE/g)	References
**Maroccon**	2.4	0.8	60.409	Kabach et al. [20]
**Germany**	2.08	1.33	48.97	Okur et al. [21]
**Tunisia**	0.035	0.025	26.8	Aouadhi et al. [8]
**Tunisia**	0.038	NA	6	Amri et al. [7]
**Algeria**	0.083	NA	40	Ouchemoukh et al. [22]
**Saudi Arabia**	0.032	0.021	36.8	Our Study

NA: not available.

**Table 3:** Acetylcholinesterase inhibitory activity of the diverse plant extract reported in different studies.

Species	AChE Activity (IC_50_ mg/mL)	References
*M. vulgare (Serbia)*	2.821	Salaj et al. [31]
*M. vulgare (Algeria)*	0.52	Ouchemoukh et al. [22]
*Marrubium desertia*	0.277	Chemsa et al. [33]
Indomethacin (control)	1	Orhan et al. [30]
*M. vulgare (Saudi)*	0.4	**Our study**

**Table 4:** Antibacterial activity of *Marrubium vulgare* methanolic extract against nine bacteria evaluated by disc diffusion and MIC and MBC tests.

Strains ^a^	Inhibition Zone Diameters (mm) ^b^		MIC (mg/mL)	MBC (mg/mL)
	Extract	Gentamicin		
**Gram-negative bacteria** *E. coli* ATCC 8739	16 ± 0.5	24	12.5	25
*S. typhimurium* NCTC 6017	15 ± 1	23	25	50
*S. arizona* ATCC 25922	14 ± 0.7	23	25	50
*P. aeruginosa* ATCC 27853	17 ± 0.8	21	12.5	25
*Klebsiella pneumonia*	18	20	6.25	12.5
**Gram-positive bacteria** *L. monocytogenes* ATCC 7644	13 ± 0.5	18	25	50
*B. cereus* ATCC1247	13 ± 0.5	21	25	50
*E. faecalis*	13 ± 0.6	19	12.5	25
*S. aureus*	15 ± 0.4	20	25	50

^a^ Final bacterial density was around 10^5^ cfu/mL. ^b^ Inhibition zone diameters (mm) produced around the wells by adding 15 µL of methanolic extract. Values are means of three measurements. ±: standard deviation; MIC: minimum inhibitory concentration; MBC: minimum bactericidal concentration.

## Data Availability

The study did not report any data.

## Sample Availability

Samples of the compounds not available from the authors.
